# The Role of MicroRNA in Paediatric Acute Lymphoblastic Leukaemia: Challenges for Diagnosis and Therapy

**DOI:** 10.1155/2019/8941471

**Published:** 2019-10-13

**Authors:** Carle Grobbelaar, Anthony M. Ford

**Affiliations:** ^1^Barts Cancer Institute, Queen Mary University of London, Charterhouse Square, London, UK; ^2^Centre for Evolution and Cancer, Division of Molecular Pathology, The Institute of Cancer Research, London, UK

## Abstract

Acute lymphoblastic leukaemia (ALL) is the most common cancer of childhood. Although the overall survival of children with ALL is now more than 90%, leukaemia remains one of the leading causes of death from disease. In developed countries, the overall survival of patients with ALL has increased to more than 80%; however, those children cured from ALL still show a significant risk of short- and long-term complications as a consequence of their treatment. Accordingly, there is a need not only to develop new methods of diagnosis and prognosis but also to provide patients with less toxic therapies. MicroRNAs (miRNAs) are small ribonucleic acids (RNA), usually without coding potential, that regulate gene expression by directing their target messenger RNAs (mRNAs) for degradation or translational suppression. In paediatric ALL, several miRNAs have been observed to be overexpressed or underexpressed in patient cohorts compared to healthy individuals, while numerous studies have identified specific miRNAs that can be used as biomarkers to diagnose ALL, classify it into subgroups, and predict prognosis. Likewise, a variety of miRNAs identify as candidate targets for treatment, although there are numerous obstacles to overcome before their clinical use in patients. Here, we summarise the roles played by different miRNAs in childhood leukaemia, focussing primarily on their use as diagnostic tools and potential therapeutic targets, as well as a role in predicting treatment outcome. Finally, we discuss the potential roles of miRNA in immunotherapy and the novel contributions made by gut miRNAs to regulation of the host microbiome.

## 1. Introduction

Leukaemia, the most common paediatric cancer, accounts for approximately one-third of malignancies diagnosed in children below 16 years of age [1]. With a peak incidence between 2 and 5 years of age, acute lymphoblastic leukaemia (ALL) accounts for 85% of leukaemia in childhood [2]. Through treatment stratification by immunophenotype and genotype, the incorporation of more effective drug combinations into treatment protocols, and improved supportive care, overall survival of patients with ALL has increased to more than 80% in developed countries [3–5].

Despite the dramatically improved survival rates achieved with most treatment protocols, leukaemia in children remains one of the leading causes of death from disease. While the diagnostic classifications allowing for personalized treatment and prognostic evaluation have greatly improved, novel biomarkers for the diagnosis and classification of leukaemia are still required [6]. In addition, short-term and long-term complications arising from treatment toxicity remain a significant risk for individuals “cured” from their disease [7]. Hence, there is also a need for the development of new therapies to effectively treat children with high-risk disease, as well as a better optimization of current treatment strategies for low-risk disease.

## 2. MicroRNA Biology and the Role in Cancer

**Figure 1:**
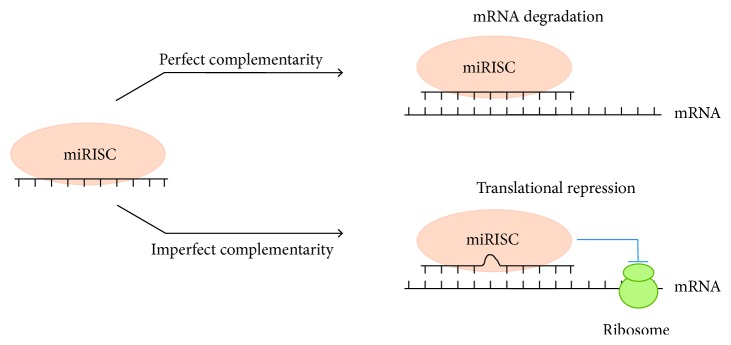
The functional mechanism of miRNA in posttranscriptional regulation of gene expression. miRNAs function to regulate the expression of genes by binding to the 3′ untranslated regions of their target messenger RNAs (mRNAs). miRNAs downregulate expression through acceleration of the degradation of mRNA (perfect complementarity) or by inhibition of its translation (imperfect complementarity).

MicroRNAs (miRNAs) in animals are single-stranded noncoding RNAs with a length of 19 to 25 nucleotides (nt), usually formed from hairpin-shaped precursors. The canonical biogenesis of miRNAs (extensively reviewed in [8, 9]) involves a complex process that converts the primary miRNA transcript (pri-miRNA, often up to 1000 nt in length) into an active mature miRNA. Briefly, the pri-miRNA transcript is processed by the Class 2 ribonuclease III enzyme, DROSHA, into a precursor miRNA (pre-miRNA, 60–120 nt) that bears a hairpin structure with a 2-nt overhang at the 3′ end. The overhang is recognised by EXPORTIN 5 (XPO5) and the pre-miRNAs are exported to the cytoplasm where they are processed by the endoribonuclease DICER into the mature miRNA duplex (19–25 nt). The 3′ or 5′ of the miRNA duplex is then loaded into the Argonaute family of proteins to form the miRNA-induced silencing complex (RISC) (Figure 1). Multiple noncanonical pathways also exist that make use of different combinations of DROSHA, EXPORTIN 5, and DICER [8]. For example, short-hairpin RNAs can be processed by DROSHA into miRNA through a DICER-independent pathway and “mitrons” can be produced from the spliced-out introns of RNA coding genes through a DROSHA-independent process. However, originally described as not having coding potential, more recent evidence has identified a subset of pri-miRNA that can indeed encode small peptides [10]. Such pri-miRNAs contain small open reading frames of around 300 nt that may be transported, unprocessed, into the cytosol where they become translated into micropeptides that can influence a variety of biological processes. Functional studies of micropeptides in humans are now beginning to show a significant association with disease and may possibly also present novel therapeutic opportunities [10]. miRNAs function by regulating the expression of genes usually through direct seed binding to the 3′ untranslated regions of their target messenger RNAs (mRNAs) and downregulate gene expression by acceleration of mRNA degradation (Figure 1). An alternative binding mechanism used by miRNAs involves particularly weak base pairing in the centre of the target pairing as a consequence of mismatched bulges in the miRNA sequence; this in turn leads to inhibition of translation [8]. miRNA binding sites have also occasionally been identified in the 5′ untranslated regions and open reading frames of mRNA, although these models are still under investigation [11]. Hence, miRNA are an important class of gene regulator estimated to be involved in the regulation of ∼30% of all genes and can regulate almost every genetic pathway. Consequently, dysregulation of miRNA expression leads to the dysregulation of downstream mRNA targets and may be accompanied by severe implications to the homeostasis of cells and tissues [12].

Similar to traditional protein-coding genes, miRNA genes can be altered by a variety of aberrations including chromosomal amplifications, transcriptional activation, deletion, methylation, and sequence variation. Alterations of miRNAs have been identified in numerous diseases such as cardiac disorders, autoimmune diseases, and cancers; in the latter, they are highly dysregulated and can act as either oncogenes (oncomiRs) and/or tumour suppressors. For example, acting as an oncogene, overexpression of miR-21 in mice accelerates development of a precursor B (pre-B) cell malignant lymphoid-like phenotype; however, tumours regress completely within a few days when miR-21 is inactivated *in vivo* [13]. The role of miRNA as a tumour suppressor is illustrated by the miR-15a/16-1 cluster, which is frequently deleted in chronic lymphocytic leukaemia (CLL) [14]. In a knockout mouse model, induced deletion of the cluster results in the development of indolent B-cell-autonomous, clonal lymphoproliferative disorders that essentially reiterate the spectrum of CLL-associated phenotypes observed in humans [14]. Furthermore, the deletion of miR-15a/16-1 accelerates the proliferation of mouse and human B-cells by modulating the genes that control cell-cycle progression [14]. Examples of miRNAs acting as an oncogene in one cell type and as a tumour suppressor in another have also been reported; miR-221 is overexpressed in liver cancers where it targets the tumour suppressor *PTEN*, thereby promoting liver tumourigenicity [15]. However, in other tumour types, for example, gastrointestinal stromal tumours, miR-221 is downregulated and the consequent downregulation of *c-KIT* and *ETV1* (its target oncogenes) results in the promotion of this malignancy [16].

### 2.1. miRNAs as General Biomarkers of Cancer

A feature of an ideal biomarker is that it must present a unique expression profile in the patient compared to healthy individuals. Biomarkers must be reliable not only for early diagnosis, preferably before the development of full clinical symptoms, but also for the detection of persistence of minimal residual disease (MRD) and the recurrence of disease after treatment. They must additionally have a long half-life in clinical samples, be accessible through noninvasive methods, and be detected through simple, accurate, and inexpensive methodology. Therefore, miRNAs demonstrate tremendous potential as biomarkers for the early diagnosis of malignancy (and its prognosis), since they can be reliably detected in and extracted from blood (total, plasma, or serum) [17]. Circulating miRNAs are packaged inside microvesicles such as exosomes [6] and their profile in patients usually reflects the pattern observed in their respective tumour tissues, making them an attractive possibility as minimally invasive and robust biomarkers [18–20]. The first reported blood-based circulating miRNA was miR-21, which showed aberrant expression levels in the serum of diffuse large B-cell lymphoma patients [6].

### 2.2. miRNA in the Diagnosis and Classification of Paediatric ALL

**Figure 2:**
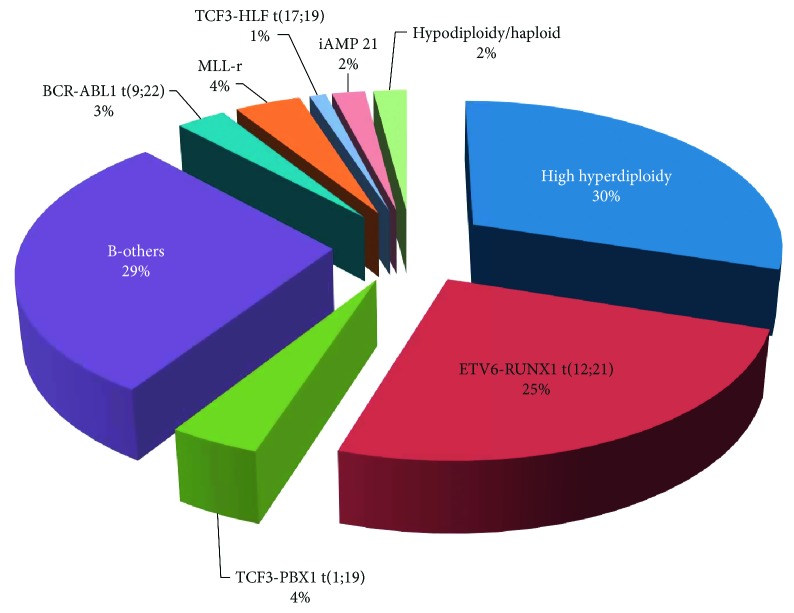
Major recurrent chromosome aberrations of childhood BCP-ALL. Hyperdiploidy and the *ETV6-RUNX1* translocation constitute the major subgroups of BCP-ALL and are associated with favourable clinical outcome. The t(1;19) and “B-Others” subgroups are associated with intermediate risk and the remaining subgroups are deemed poor risk.

ALL and acute myeloid leukaemia (AML) can be distinguished by a variety of morphological, immunophenotypic, and immunohistochemical methods; nonetheless, few single tests are currently sufficient for the establishment of diagnosis. The current classification of ALL is based on cell morphology, immunophenotypic characteristics, and cytochemical, cytogenetic, and molecular features, and can be divided into two distinct subgroups: B-cell precursor ALL (BCP-ALL) and T-cell ALL (T-ALL). BCP-ALL can be further subclassified according to recurrent genetic abnormalities [21, 22] and Figure 2.

To address this issue, Mi and colleagues [23] performed large-scale genome-wide miRNA expression profiling and identified 27 miRNAs that are differentially expressed between ALL and AML. Of these, miR-128a and miR-128b were significantly overexpressed in ALL compared to AML, whereas lethal (let)-7b and miR-223 were significantly downregulated. Their results show that ALL can be discriminated from AML with an accuracy of greater than 95% based on the differential expression pattern of these four miRNAs [23].

**Figure 3:**
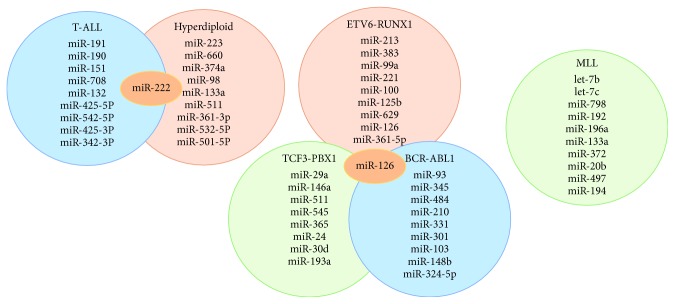
A top-ten discriminative miRNA set for the main leukaemia subgroups. The subtypes display a unique discriminating miRNA (except where overlap is shown) that distinguishes each subgroup from each other [25].

Certain miRNA profiles have also been shown to associate specifically with paediatric ALL [24] and reveal distinct expression profiles based on the cell of origin and cytogenetic subtype. BCP-ALL was distinguishable from normal bone marrow (BM) and CD34+ cells by presenting a significantly lower expression of miR-127 and miR-143. Similarly, T-ALL cells differed extensively from normal thymocytes with differential expression of 28 miRNAs. In high hyperdiploid ALL, high expression of a variety of miRNA was also observed [24]. Interestingly, except for *BCR-ABL1*-positive and “B-other” ALL, all the subtypes display a unique miRNA signature that distinguishes each group from all the other subtypes. A top-ten discriminative miRNA set was proposed for each subgroup as shown in Figure 3 [25].

Significantly different levels of expression of miR-100 and miR99a have been observed in childhood ALL, with very low levels of expression seen in patients with ALL compared to those with AML or in healthy BM donors [26]. Conversely, an association between increased miR-100 levels and the presence of t(12;21)-positive ALL suggested the possibility of a t(12;21)-specific regulation of miR-100 [27]. Expression levels of two additional tumour suppressor miRNAs, miR-326 and miR-200c, appear to be significantly downregulated in BM mononuclear cells of paediatric ALL patients at diagnosis. Moreover, area under the curve (AUC) in receiver operating characteristic curve analysis (ROC) showed a high sensitivity and specificity for both miRNAs in discriminating between paediatric ALL patients and healthy individuals. These findings suggest that miR-326 and miR-200c may be involved specifically in leukaemogenesis and serve as potential reliable noninvasive diagnostic biomarkers of paediatric ALL [28].

RT-qPCR analysis of expression levels of miR-203 and miR-125b in peripheral blood (PB) isolated from 43 newly diagnosed children with ALL showed the median level of miR-125b expression to be 33-fold higher in the ALL cases than in the healthy control group. On the contrary, the median expression level of miR-203 was 31-fold higher in the control group when compared to the ALL cases. While the sensitivity of miRNA-203 was higher than that of miRNA-125b, their combination revealed absolute sensitivity, suggesting that preclinical studies targeting miRNAs for diagnosis of ALL should be strongly encouraged [29].

Disruption of the function of miR-181a, a regulator of normal haematopoiesis, has been associated with many cancer types. Depending on the cellular context and on the consequent expression of its targets, miR-181a can act as either a tumour suppressor or an oncogene. Nabhan and colleagues [30] investigated the expression levels of miR-181a in 30 newly diagnosed paediatric ALL patients, comparing these results with 30 sex-matched normal healthy children as the control group [30]. Serum samples of the ALL group showed a highly significant decrease in expression levels of miR-181a compared to those found in the healthy children. *ETV6-RUNX1*, the fusion product of t(12;21), is the most common genetic abnormality observed in paediatric ALL and especially BCP-ALL [31, 32]. Consistent with a study that confirmed that miR-181a is able to effectively target the fusion, decrease its protein level, and induce a significant antileukaemic effect, it was noted that miR-181a plays a tumour suppressor role in ALL [30, 33]. Furthermore, miR-181a was identified as the most differentially downregulated miRNA in the PB of paediatric ALL patients that express ETV6-RUNX1 [34].

Additional investigations were performed to evaluate the expression levels of the nuclear protein “mothers against decapentaplegic homolog 7” (SMAD7), a miR-181a target pair, and transforming growth factor-b1 protein (TGF-*β*1), the response of which is negatively regulated by SMAD6 and SMAD7 [30]. Results showed that SMAD7 protein levels were significantly higher in ALL patients compared to the healthy control group, whereas TGF-*β*1 protein was significantly lower. Of note, deregulation of TGF-*β*1 signalling through SMAD7 overexpression has been previously associated with the pathogenesis of ALL [35]. With the combined use of miR-181a and SMAD7, the sensitivity of diagnosis was increased to 90%, whereas the combined use of miR-181a and TGF-*β*1 increased sensitivity to 100%. These data suggest that the diagnostic accuracy of paediatric ALL can be improved by using a small combination of biomarkers [30].

### 2.3. miRNA in the Prognosis of Paediatric ALL

Paediatric ALL is currently stratified according to different biological and clinical parameters with patients subsequently receiving risk-adapted therapy. Albeit current strategies yield a very high cure rate, there are a number of patients with ALL who will ultimately relapse. The classical use of both gene expression profiling and mRNA signatures in the clinical setting has disadvantages and only intermediate success has been reported. Since miRNA profiling is usually limited to a small number of genes, it can potentially offer fewer and more robust signatures that have an equally strong prognostic capacity [25].

Expression levels of miR-16, a gene lost in many cases of CLL, were assessed in 93 children with ALL and results associated to the main biological and clinical variables that define prognosis: white blood cell (WBC) count, age at diagnosis, cyto- and molecular-genetic profile, and risk groups. While the highest expression values of miR-16 associated with a poor prognosis, low levels associated with a good outcome [36]. The disease-free survival (DFS) and overall survival (OS) were evaluated according to miR-16 expression profiles in the ALL group as a whole and in the B-cell and T-cell ALL subgroups. Group analysis showed that DFS was longest for patients with miR-16 expression less than quartile 25 and the shortest for those patients with miR-16 expression above quartile 75. Relapse was rarely observed in the patients with low miR-16 levels, while the shortest DFS was in the quartile above 75. Similarly, in the T-ALL group, patients in the quartile above 75 presented the shortest survival, whilst the longest survival was observed in the quartile below 25. Statistically significant relationships were also obtained between low miR-16 levels and low WBC and good cyto- and molecular-genetic markers [36].

**Figure 4:**
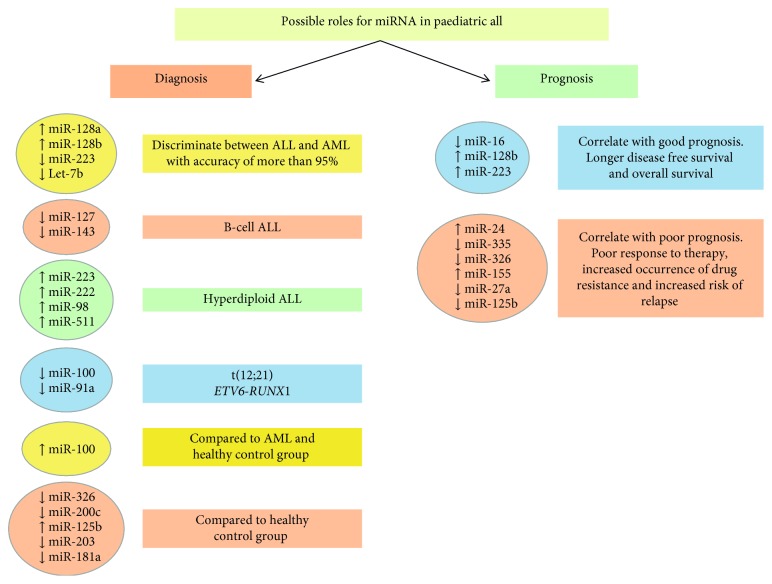
Potential applications of miRNA as a diagnostic and prognostic tool in paediatric ALL. The figure summarises those miRNAs that are either upregulated or downregulated in the various subsets of ALL and their potential use as a tool for its diagnosis and prognosis.

In addition, a multivariate analysis of miR-24 expression, including age at diagnosis, gender, and WBC, defined miR-24 as an independent prognostic marker. Upregulation of miR-24 was associated with a significant shorter OS, compared to those cases with low expression [37]. These results strongly suggest that high miR-24 expression levels could be used as a reliable and effective biomarker of poor prognosis ALL (Figure 4).

The changes in miRNA expression in relapsed ALL are the focus of several research groups and suggest that miR-223 and miR-128b levels could be possible predictors of ALL relapse. Nemes and colleagues [38] collected PB and BM samples from 24 ALL patients from all phases of treatment. They determined that whilst miR-223 expression was almost undetectable at diagnosis, the levels of expression recovered to normal during treatment and in remission, but then decreased again at relapse. Conversely, an extreme overexpression of miR-128b was observed at diagnosis, which significantly decreased as patients entered remission where normal levels of miR-128b expression for mononuclear cells were detected. Higher miR-128b expression levels at diagnosis also correlated with a better prognosis, suggesting that monitoring of miR-128b expression levels in PB could assist in the early detection of disease relapse (Figure 4) [38].

### 2.4. miRNA in Treatment, Treatment Response, and Toxicity of Paediatric ALL

Most children diagnosed with ALL follow a standard treatment protocol as part of a risk-adapted strategy that is typically divided into 3 main phases: induction, consolidation, and maintenance, and a fourth phase of CNS-directed therapy. Although in recent times the main cytotoxic agents used in the induction phase of treatment have not changed, they usually include combinations of drugs including prednisolone (PRED), vincristine (VCR), L-asparaginase (L-ASP), and daunorubicin (DNR). Multidrug regimens are used in the attempt to prevent drug resistance but all agents have undesirable late effects. Maintenance therapy, the final and most prolonged treatment in childhood ALL, involves a much less intensive regimen than the prior chemotherapy. Lasting up to two years, the maintenance phase has been demonstrated to lower the risk of relapse but may itself be a cause for the emergence of new (drug resistant) mutations.

### 2.5. miRNA and Multidrug Resistance

Multidrug resistance (MDR) is a ubiquitous and severe clinical problem in the treatment of paediatric ALL and is often mediated by adenosine triphosphate (ATP)-binding cassette (ABC) transporters. Various substrates, such as drug molecules, are actively transported by ABC transporters across extracellular and intracellular membranes, resulting in the restriction of drug deposition and chemotherapy failure [39]. A significant amount of evidence now shows that miRNAs tend to impact upon therapy response through modulation of expression levels of a variety of proteins, including ABC transporters [40]. Transfection of resistant HepG2 cells with miR-326 leads to enhanced sensitivity to chemotherapeutic drugs due to the downregulation of ABC transporter ABCC144. Furthermore, overexpression of ABCA2 and ABCA3 genes significantly increases the risk of MDR and relapse in children with ALL [41]. Bioinformatics analysis shows miR-326 to be a negative regulator of drug resistance-related genes, particularly ABCA2 and ABCA3 [27]. Evaluation of the expression levels of miR-326 in paediatric ALL patients resistant to chemotherapy revealed a significant decrease in miR-326 expression levels in MRD+ patients compared to the MRD- group, supporting the notion that downregulation of miR-326 has an adverse impact on chemotherapy treatment response [28]. The downregulation of miR-326 in children with relapsed ALL compared to the expression levels observed at first diagnosis, indicate that miR-326 can be considered another prognostic marker for relapse in paediatric ALL (Figure 4).

To identify novel prognostic and therapeutic predictors in ALL, a genome-wide miRNA expression study was performed on 63 newly diagnosed childhood ALL patients [42]. The miRNA profiles of the relapse/deceased cases were compared to those in complete remission (CR), identifying miR-335 as the most significantly downregulated miRNA. miR-335 gene promoter regions reveal significant methylation patterns in relapsed patients, suggesting that miR-335 is epigenetically silenced through DNA methylation. Epigenetic silencing of miRNA genes is associated with clinical outcome in infant ALL [43, 44]. Significantly poorer 5-year event-free survival was observed in patients with low expression levels of miR-335, and a multivariate analysis confirmed miR-335 levels at diagnosis to be an effective and independent prognostic marker in predicting treatment outcome [42].

miR-155 is believed to play a role in inhibition of lineage differentiation [45], holding haematopoietic stem and progenitor cells (HSPCs) at the early stem cell stage. El-Khazragy and colleagues [46] determined whether miR-155 expression levels correlate with clinic-pathological features of the disease; the effect of therapy on the expression levels of miR-155 was also evaluated by RT-qPCR. BM samples of 45 children with ALL were collected at diagnosis to determine miR-155 expression levels, and percentage of blasts postinduction was assessed on day 15 as a measure of MRD. Additionally, expression levels were determined after treatment in samples taken on day 28 and results compared with a control group of ten healthy controls. A significant high level of miR-155 expression was detected at diagnosis in the ALL group, and a significant correlation was observed between high miR-155 levels and high blast numbers (>25%), unfavourable cytogenetic abnormality, total WBC, higher relapse rate, and a higher MRD after 15 days. The overexpression of miR-155 therefore correlates with poor prognosis in paediatric ALL, due to a decrease in the response to therapy and an increase in relapse. Furthermore, miR-155 expression levels were significantly decreased 10-fold after therapy, again suggesting potential use as a biomarker of therapeutic response in childhood ALL (Figure 4) [46].

To further determine the miRNA expression profiles and miRNA patterns associated with childhood ALL relapse, a genome-wide miRNA microarray analysis was performed on paired diagnostic-relapse and diagnostic-CR series of paediatric ALL cases [47]. A differential miRNA pattern was identified between relapse and CR patients that included miR-223 and miR-27a: both miRNAs were highly expressed in patients during CR. Furthermore, the expression levels of both miR-223 and miR-27a were low in diagnostic samples of patients who subsequently relapsed during the study. miR-223 expression at initial diagnosis is another independent and reliable predictor for OS in paediatric ALL patients [47].

Piatopoulou and colleagues [48] examined miRNA profiles at diagnosis and after induction, comparing those levels with known prognostic features of ALL. Lower miR-125b expression levels at diagnosis and higher levels at the end of the induction protocol were associated with adverse disease features. The stronger risk for short-term relapse and a worse OS were clearly demonstrated by survival analysis of patients who presented with underexpressed miR-125b at diagnosis and overexpression after induction [48]. Albeit miR-125b presented with a wide variability of expression at diagnosis, lower expression levels correlated with unfavourable prognostic features including high-risk age group, high BM blast count at diagnosis and day 15 after induction, as well as patients with a low haemoglobin concentration. Downregulation of miR-125b is also significantly associated with higher risk of relapse and a poor OS, providing convincing evidence that miR-125b could also be used as a potential prognostic biomarker in paediatric ALL [49].

**Figure 5:**
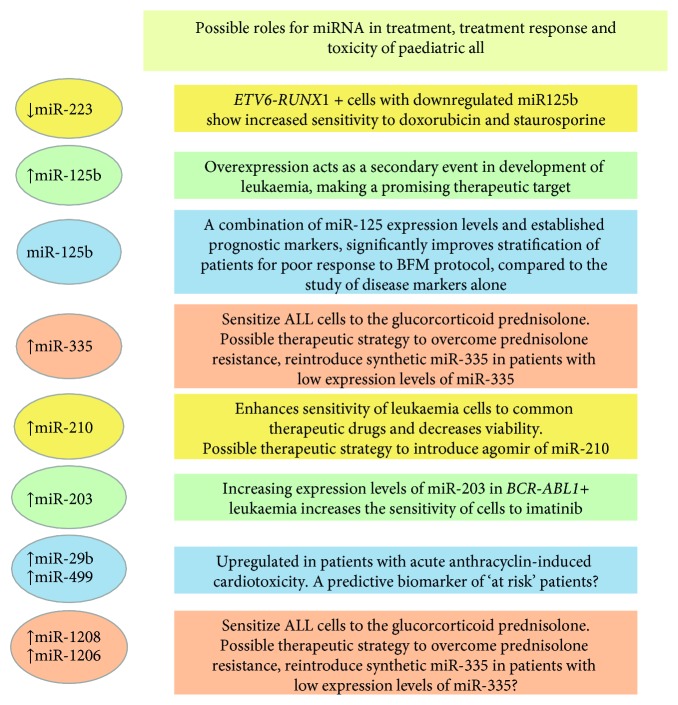
Potential applications of miRNAs as biomarkers of treatment response and toxicity in paediatric ALL. The figure summarises those miRNAs that are either upregulated or downregulated in the various subsets of ALL during therapy. Their potential use as a tool for treatment or marking treatment response is discussed in the text.

With the development of miRNA inhibitors and mimics, along with antagomiRs and agomiRs, and the possible reexpression of miRNAs by delivery of their precursors, it is now becoming feasible to restore miRNA levels to normal, at least *in vitro*. A very attractive and possible future treatment strategy will therefore be to combine miRNA-based therapy with conventional chemotherapy. Gefen and colleagues [50] established that the expression of the miR-125b-2 cluster, consisting of miR-125b, miR-99a, and let-7c, is increased in *ETV6-RUNX1+* leukaemia. Through manipulation of *ETV6-RUNX1* gene expression and chromatin immunoprecipitation, their results show that the miRNA-125b-2 cluster is not regulated by the ETV6-RUNX1 fusion protein itself, suggesting that expression of this cluster could be an independent leukaemia event. Knockdown studies of endogenous miR-125b in the *ETV6-RUNX1+* cell line Reh increased cell sensitivity to doxorubicin and staurosporine treatment. Furthermore, overexpression of miR-125b-2 conferred a survival advantage through the inhibition of apoptosis and activation of caspase-3 [50]. These results suggest that miR-125b-2 cluster is another potential therapeutic target in paediatric ALL (Figure 5).

The *in vitro* sensitivity of cells isolated from patients with BCP-ALL following standard treatment with prednisolone (PRED), vincristine (VCR), L-asparaginase (L-ASP), or daunorubicin (DNR) has been studied for respective changes in miRNA profiles. While only one miRNA, miR-454, was linked to L-ASP resistance and none to prednisolone, resistance to VCR and DNR was characterized by a 20-fold upregulation of miR-125b, miR-99a, and miR-100. Moreover, 39% of the patients resistant to VCR were of *ETV6-RUNX1* subtype; the *ETV6-RUNX1+* patients and the VCR-resistant cases both presented higher miR-125b expression levels. The overexpression of miR-125b reduced the amount of drug-induced apoptosis in pre-B-cells and induced the proliferation of CD34+ cells, suggesting that the interference of miR-125b function might provide a way to sensitize patients to these chemotherapeutic drugs [24]. The potential role of miR-125b in paediatric ALL treatment has now been studied *in vivo* by Bousquet and colleagues [51]. Twenty mice were transplanted with miR125b-overexpressing foetal liver cells and of these, half succumbed to a haematological malignancy within 12 to 29 weeks post transplantation. The phenotypes included both BCP-ALL and T-ALL, suggesting that miR-125b may play a role in the differentiation process of both lymphoid lineages. The authors conclude that miR-125b, as a driver mutation or secondary event, could also provide a promising therapeutic target [51]. Furthermore, miR-125b overexpression was shown to accelerate the oncogenicity of the BCR-ABL1 fusion protein, a hallmark of chronic myeloid leukaemia (CML) but one also observed in a subset of paediatric ALL [52]. To evaluate the clinical utility of miR-125b for patients treated on the Berlin-Frankfurt-Münster (BFM) protocol, BM samples of childhood ALL patients were obtained at diagnosis and day 33 and compared to healthy controls. Whilst miR-125b expression levels were downregulated at diagnosis, a significant overexpression (between 2- and 5-fold) was observed on day 33 [48]. To explore a role for miR-125b, miR-99a, and miR-100 in therapy resistance, Reh cells were treated with VCR for 3 days [53]. The baseline expression levels of these 3 miRNAs in Reh cells were in the range of levels observed in the leukaemic cells of children with *ETV6-RUNX1+* ALL. Results showed that miR-125b, in combination with miR-99a or miR-100, induced resistance to VCR, whereas the same effect was not observed for each of the miRNAs individually. After combined overexpression of miR-125b/miR-100, miR-125b/miR-99a, or miR-125b/miR-99a/miR-100, the fold change in VCR resistance did not differ. The mature sequences of miR-99a and miR-100 differ only by one nucleotide and consequently have considerable overlap in the list of their predicted target genes. The rate of apoptosis and cell-cycle distribution in the absence of VCR did not vary between Reh cells expressing miR-125b together with miR-99a and/or miR-100, compared to those cells expressing miR-125b in combination with a scrambled miRNA control. These results suggest that for the development of VCR resistance, the combined overexpression of miR-125b, miR-100, and/or miR-99a may provide a specific trigger. Whether the target genes directly regulated by these miRNAs and subsequent effected pathways could provide a way in which VCR resistance in *ETV6-RUNX1+* ALL could be modulated remains to be seen [53].

Glucocorticoids (GCs) are also widely used in the clinical treatment of paediatric ALL; the interindividual differences in GC therapy response, however, compromise their clinical application. Additionally, GC resistance is one of the major contributing factors of poor outcome in paediatric ALL. Recent studies have shown that miRNAs play a crucial role in GC sensitivity and may provide potential strategies to overcome GC resistance [54]. In a genome-wide miRNA analysis of paediatric ALL patients, a reduced miR-335 expression was identified as the most significant miRNA abnormality associated with poor outcome [42]. Overexpression of miR-335 significantly sensitized the ALL cells to PRED, across a range of concentrations, indicating that ectopic expression of miR-335 can confer an enhanced sensitivity. Since no enhancement of cell death was observed in cell lines overexpressing miR-335 when treated with other chemotherapy drugs, it was proposed that miR-335 downregulation could decrease the susceptibility of ALL cells to PRED treatment. To determine the functional role of miR-335 in PRED resistance, investigation of downstream pathways revealed that a low level of miR-335 results in higher MAPK1-mediated survival. Moreover, MEK/ERK inhibitor treatment enhanced PRED-induced cell death suggesting that reintroducing synthetic miR-335 and overriding MAPK1 activity and MEK/ERK pathway inhibition could provide a basis for the development of further therapeutic strategies to overcome PRED resistance and so improve treatment outcome in ALL patients [42].

Clinical studies indicate that miR-210 is differentially expressed in several types of cancer. To establish its effect on treatment outcome in leukaemia, intracellular miR-210 levels were altered by transfecting two different paediatric ALL cell lines (*ETV6-RUNX1+* Reh and *MLL-AF4+* RS4;11) with an agomiR and antagomiR to miR-210 [55]. Twenty-four hours after transfection, both cell lines were treated for 48 hours individually with the four common therapeutic drugs (dexamethasone [DEX], VCR, DNR, and L-ASP) or in four drug combinations. The miR-210 agomiR produced an approximate 30-fold increase and antagomiR an 80% decrease, respectively, compared to the negative controls. In Reh cells, the half maximal inhibitory concentrations (IC50s) of DNR, DEX, and L-ASP were significantly decreased (agomiR) or increased (antagomiR) compared to those cells transfected with the negative control mimics. Similarly, in RS4, 11 cells, the IC50s of DNR, DEX, and VCR (but not L-ASP) were decreased and increased by the miR-210 agomiR and antagomiR, respectively. Based on these results, the use of agomiRs or antagomiRs of miRNAs could be a feasible alternative to overcome resistance to chemotherapy. It was, however, noted that safe and effective delivery of synthetic miRNAs to leukaemia cells still remains a great challenge [55]. As discussed later, it will also be paramount to study the off-target effects of using particular miRNA as a therapeutic target, either alone or with chemotherapy, since both treatments can regulate the expression of other critical miRNA.

Although quite rare in children, ALL patients that harbour the p190 variant of the *BCR-ABL1* (Ph+) fusion gene are treated with tyrosine-kinase inhibitors (TKI) such as imatinib, but the prognosis of these patients remains suboptimal. *ABL1* is a direct target of miR-203 and in malignancies expressing either *ABL1* alone or *BCR-ABL1*, miR-203 is silenced through genetic and epigenetic mechanisms. Restoration of the expression of miR-203 reduces levels of *ABL1* and *BCR-ABL1* and inhibits cell proliferation [56, 57]. To determine whether the inhibition of *BCR-ABL1* by miR-203 is sufficient to overcome resistance to TKI, cells expressing *BCR-ABL1*, as well as cells overexpressing both *BCR-ABL1* and miR-203 together, were treated with imatinib and cell viability determined. At all drug doses, the *BCR-ABL1+* cells without miR-203 overexpression retained higher viability, suggesting that the sensitivity of *BCR-ABL1+* cells to imatinib could be increased with the restoration of miR-203 [58].

### 2.6. miRNAs as Tools for Immunotherapy

In the main, studies of miRNA biology have focused on their role as either oncogenes or tumour suppressors; however, recent advances in our understanding of the immune system suggest that miRNA also play an important role in immune regulation and can therefore act as both agents and targets of immunotherapy. Immune checkpoints play a significant role in cancer therapy; control of checkpoint receptors through miRNA may be through direct target of checkpoint genes or via other proteins that themselves are regulated by miRNA. Checkpoint inhibitors are able to block the proteins that prevent the immune system from attacking cancer cells. As discussed above, in the context of being able to restore normal miRNA levels, miRNA mimetics can be used to restore the expression of the various downregulated miRNAs that target immune checkpoint mRNAs and consequently can serve to reduce the levels of checkpoint proteins. A comprehensive role for miRNA in B-cell biology is rapidly being documented, but one important established mark is in the acceleration of bone marrow differentiation. Numerous miRNA appear expressed in a stage or disease-specific fashion in both early and late B-cells, suggesting functional and/or pathologic specificity. Koralov and colleagues [59] have shown that a deficiency in Dicer results in a significant defect in B-cell differentiation at the pro-B to pre-B-cell stage, and a number of miRNAs have been identified that are dynamically controlled during early B-cell lineage specification [60]. For example, the expression level of miR-155 is raised in murine BCP-ALL, suggesting that miR-155 can cause arrest and accumulation of pre-B-cells [61]. *Programmed cell death protein 1* (PD1) is a cell surface receptor checkpoint protein expressed on pro-B-cells (and T-cells) that down regulates the immune response [62]. The antitumor activity of PD-1 immune checkpoint blockade has been established in clinical trials [63] where solid tumour regression was observed. Targeting *PD-1*, or the genes of its ligands (*PD-L1* and *PD-L2*), has since identified a number of miRNAs (including miR-34a and miR-424) that can activate anticancer immunity, initiate apoptosis, and reverse chemoresistance by blocking the PD-1 immune checkpoint [64, 65]. In addition, miR-142-5p was found to downregulate the expression of PD-L1 by directly binding to the 3′ UTR of *PD-L1* mRNA [66]. The let-7 family of miRNA is generally considered to be a good biomarker for the diagnosis and prognosis of ALL, but have also been shown to be involved in metabolic control and the normal immune response [67]. As discussed later, one exciting prospect for future treatment strategies is the knowledge that the gut microbiome in patients for whom PD-1 blockers work well differs considerably from those patients in whom they fail [68].

Chimeric antigen receptor-T (CAR-T) cell therapies have recently begun to show dramatic success in clinical trials that treat patients with BCP-ALL [69]. Analysis of the transcriptome profiles in patients with B-ALL before and after CAR-T therapy, in combination with miRNA-seq, revealed that many miRNAs (including let-7 family members) are involved in the immune process and could also regulate the crosstalk between the transcription factors and histone proteins involved in the response to CAR-T therapy [70]. For example, within an elegant regulatory network, the authors showed that FOS, JUN, and CEBP regulate the expression of histone genes *HIST1H4A* and *HIST2H4A*, which were targeted by miR-148a-3p. In addition, 15 upregulated and 7 downregulated miRNAs were also identified in patients in remission that may now become additional biomarkers of prognosis.

### 2.7. miRNA and Late Effects of Treatment

Anthracyclines such as doxorubicin are also used widely in the treatment of paediatric leukaemia. Anthracycline (AC)-related cardiotoxicity is one of the most significant long-term threats to survivors of cancer, with cardiac events being the most common cause of death in this population. It has been estimated that the prevalence of cardiomyopathy in 50-year-old survivors of childhood cancer exposed to cardiotoxic chemotherapy is 21% [71]. AC-induced cardiomyopathy is progressive, with no definitive treatment, and currently methods to detect cardiac injury early in the course of its progression are lacking. The specificity and sensitivity of detection that has been established by miRNAs in various cardiovascular disease states indicates a potential role for miRNAs in early detection of cardiotoxicity. To identify AC-induced alterations in the expression of plasma miRNA and to correlate these changes to known markers of cardiac injury, a prospective cohort study was performed on children receiving AC chemotherapy [71]. A key finding was that the overall dysregulation of a panel of plasma miRNAs (with cardiac relevance) was greater following AC therapy compared to the control group. Furthermore, plasma miR-29b and miR-499 were particularly upregulated in patients as demonstrated by increased troponin concentrations post-AC. The post-AC expression levels of miR-29b and miR-499 correlate significantly with cumulative AC dose, a known predictor of cardiotoxicity risk. Further studies are required to determine the mechanistic role of these miRNAs in AC-induced cardiac injury but miR-29b and miR-499 could be useful for identifying patients at high risk for developing AC-induced cardiomyopathy and subsequently in the early detection of cardiomyocyte injury [71].

Methotrexate (MTX), widely used in the consolidation phase of treatment for childhood ALL, is also considered to be a main cause of the hepatotoxicity observed in two-thirds of patients. Many genes involved in normal liver-specific signalling pathways are tightly controlled by miRNAs and shown to be open to modulation by variations in miRNA gene sequence. A significant association between high levels of transaminase toxicity during MTX treatment in the consolidation phase (but not induction phase) and the presence of a single nucleotide variation (rs264881) that effects stability in the pre-miRNA of miR-1208 has been identified [72]. As expected, targets of miR-1208 include genes of the MTX pharmacodynamic and pharmacokinetic pathways; among them, dihydrofolate reductase (*DHFR*) is a principal target of MTX. This finding suggests that a higher expression of miR-1208 could perhaps moderate the adverse toxic effects of MTX that arise through inhibition of DHFR [72]. Other similar miRNA-related SNPs have shown their worth as tools for the prediction of treatment-related toxicity; for example, more than one study has confirmed the association between the rs2114358 variant in miR-1206 and MTX-induced oral mucositis, which occurs in 20% of MTX-treated paediatric ALL [73, 74]. The search continues to discover other beneficial miRNA with nucleotide sequence variations that may also become predictors of toxicity. To date, no miRNA drug candidates have entered into phase 3 clinical trials for the treatment of patients with ALL, and the long-term effects of such treatments remain to be evaluated. However, off-target effects of miRNA antagonists and mimics may trigger both neurotoxicity and immunotoxicity [75].

### 2.8. Future Perspectives: Gut miRNA

Implicated in host metabolism, immunity, and disease, the gut microbiota are a complex and diverse population of commensal bacteria, the composition of which in children is influenced by genetics, birth route, diet, and disease [76]. There is a significant interpersonal and interspecial variation between the gut microbiota of individuals, characterized by a balance and composition that is usually unique to and beneficial to the host. Disruption of this balance results in disease susceptibility; therefore, it is important to characterize the biological mechanisms through which the host can maintain the gut microbiota composition and to understand how this relationship is affected during pathological changes.

Recent studies have identified host gut or faecal miRNAs as a readily detectable and quantifiable normal component of human faeces [76]. Whilst the functional role of miRNAs in the communication between the host and microbes is only just beginning to be understood, recent findings indicate that miRNAs produced by the host intestinal epithelial cells can affect bacterial growth within the gut and hence participate in shaping the gut microbiota. Indeed, miRNA can enter bacteria and regulate bacterial gene expression and growth [76]. Furthermore, it has been shown that host miRNA expression levels can become dysregulated in the presence of a disturbed gut microbiota (for example, after chemotherapy or antibiotics) and that expression of host miRNA can in turn be influenced by the gut microbiota [77, 78]. A link between the gut microbiota and miRNA expression has been well studied in colorectal cancer (CRC) and confirms that certain miRNAs can mediate an interaction between the host and the microbiome. Novel mechanisms have also been implicated whereby miRNA may provide a possible target for therapeutic strategies in CRC patients [79]. Furthermore, numerous studies have investigated how faecal miRNAs can be exploited as potential noninvasive diagnostic and prognostic biomarkers. The faecal-based miR-20a and miR-221 are only two of the several faecal miRNAs that have so far been identified as promising diagnostic biomarkers of CRC [80, 81]. Adult survivors of childhood BCP-ALL often have health problems that continue for many years after the end of treatment, and chemotherapeutic agents and antibiotics clearly have an adverse effect on the gut and associated tissues. Reduced microbial diversity evidently exists in survivors of BCP-ALL and may be accompanied by an increase in biomarkers of inflammation (such as IL-6) and the activation of T-cells [82]. High levels of IL-6 are thought to play a role in expression of let-7a [83]. In accord with the “Greaves hypothesis” of an infectious origin for BCP-ALL [82], Bürgler and Nadal [84] suggest that memory T-helper (Th) cells may be attracted by preleukaemic B-cells and can then be activated through presentation of antigens. BCP-ALL cells can also respond to Th cell cytokines [84]. Correspondingly, miRNA have been shown to play a role in the regulation of cytokine genes and to be strongly regulated themselves during inflammation. Indeed, many downstream components of TGF-*β* signalling, eg, *SMAD* genes, contain miRNA binding sites and SMADS can induce positive or negative regulation of miRNA transcription [85]. SMAD3 has been shown to play a role in dysregulation of the TGF-*β* pathway in *ETV6-RUNX1+* preleukaemia [86] and may contribute to both the persistence or maintenance of covert preleukaemic clones in healthy children and enhance their competitive positive selection in an inflammatory context. Determination of the “preleukaemic” gut miRNA profile in the faeces of healthy children with *ETV6-RUNX1* fusion remains an important goal.

There is now substantial evidence that the normal gut microbiome is acquired very early in life, particularly in the first year, and can be modified for up to three years, where it finally stabilises [87, 88]. The evidence that microbiome status is a key risk variable in ALL is indirect and based upon indirect measures made through epidemiological studies [89]. However, the important acquisition of the microbiome in early life involves the same features implicated in risk of BCP-ALL: birth route, breastfeeding, and social contacts. Microbial exposures earlier in life appear protective but, in their absence, later infections may trigger the critical secondary mutations and gene deletions necessary for overt leukaemia. Moreover, BCP-ALL has a worldwide incidence that tracks with socioeconomic development and there is now considerable evidence to indicate that children in developed countries have a gut microbiome that is lower in diversity from those living in more basic environments [89]. Currently, several groups are performing studies to determine the expression levels and functional role of faecal miRNA and gut bacteria in childhood leukaemia. Information obtained will provide insight into the pathogenesis of childhood ALL and intrinsically particular miRNA may be considered as tools for prevention of certain subtypes of ALL. It is currently unknown, in the context of ALL, which bacteria or viral species are most relevant to neonatal immune priming and which, if any, might have the capacity to prevent, or provide the infection trigger, for overt ALL to develop from a clinically silent preleukaemia. One way to assess whether bacterial species and relevant host miRNA are critical to either prevention or promotion of ALL will be to assess in young children the impact of antibiotics on erosion of the beneficial gut bacterial microbiome. Faecal miRNA transplantation has already been shown to be able to restore the gut microbiota [76] and will undoubtedly provide future therapeutic options. In humans, these biomarkers may help to provide candidate species for intervention with prebiotics and probiotics.

## 3. Conclusions

miRNAs, a class of noncoding RNAs, target mRNAs and regulate gene expression posttranscriptionally. Furthermore, miRNAs are differentially expressed in distinct stages of lymphopoiesis and influence the maturation process of lymphoid precursors. The aberrant miRNA signatures observed in ALL can be used to define biomarkers for diagnosis, classification, and prognosis of this disease. Circulating miRNAs can be detected with the use of sensitive and easily applicable methods such as RT-qPCR, allowing for easy detection and a minimally invasive approach for diagnosis of ALL. Indeed studies are currently being performed to establish a highly sensitive and specific set of 2‐3 miRNAs that will allow for accurate diagnosis and classification of this haematological malignancy [90]. Specific profiles of miRNA expression have also been described for commonly used drugs, uncovering miRNAs that are associated with treatment response. Changes in expression levels of several miRNAs have been shown to play functional roles both in leukaemogenesis and in drug resistance; reversal of such expression profiles could improve drug sensitivity and subsequently give rise to better clinical outcomes [29, 91]. A number of miRNA have consistently been reported to be dysregulated in paediatric ALL, incorporating different cellular or molecular subgroups [27, 92]. Whilst the utilization of miRNA as diagnostic and predictive biomarkers is promising, there are still a few difficulties that need to be addressed [91]. There are inconsistencies in the methods being used for miRNA detection between different studies and comparable studies may share limited similarity within mRNA profiles when different stages of normal and aberrant cells are compared. There is therefore a need for uniformity in the collection of cells in both the experimental and control groups, as well as standardisation in the methods of detection [91, 93]. Currently, there are limited strategies to interrupt miRNA function; whilst the transfection of miRNA mimics or miRNA inhibitors *in vitro* allow for the increase or decrease of specific miRNA expression levels, safety concerns and degradation effects still limit their efficacy *in vivo*. The need for systemic delivery of miRNA as a therapeutic agent in the treatment of ALL itself raises the issue of unforeseen late effects of treatment.

Finally, albeit that survival of children with ALL has dramatically improved, there is still a need for defining novel sensitive, efficient, and reproducible biomarkers such as miRNA that can be used for early diagnosis, classification, prediction of treatment response, and ultimately perhaps even its prevention.
